# Expression of MICA in Zero Hour Biopsies Predicts Graft Survival After Liver Transplantation

**DOI:** 10.3389/fimmu.2021.606146

**Published:** 2021-07-20

**Authors:** Thomas Resch, Hubert Hackl, Hannah Esser, Julia Günther, Hubert Schwelberger, Paul Viktor Ritschl, Susanne Ebner, Manuel Maglione, Vanessa Mellitzer, Matthias Biebl, Robert Öllinger, Heinz Zoller, Stefan Schneeberger, Katja Kotsch

**Affiliations:** ^1^ Department of Visceral, Transplant and Thoracic Surgery, Center of Operative Medicine, Medical University of Innsbruck, Innsbruck, Austria; ^2^ Institute of Bioinformatics, Biocenter, Medical University of Innsbruck, Innsbruck, Austria; ^3^ Department of Surgery, Charité-Universitätsmedizin, Berlin, Germany; ^4^ Department of Medicine I, Gastroenterology, Hepatology and Endocrinology, Medical University of Innsbruck, Innsbruck, Austria; ^5^ Department of General- and Visceral Surgery, Charité–Universitätsmedizin Berlin, Corporate member of Freie Universität Berlin, Humboldt-Universität zu Berlin, Berlin, Germany

**Keywords:** biomarker, liver transplantation, graft quality assessment, graft survival, marginal donor, donor risk index, mortality

## Abstract

In search for novel biomarkers to assess graft quality, we investigated whether defined candidate genes are predictive for outcome after liver transplantation (LT).

Zero-hour liver biopsies were obtained from 88 livers. Gene expression of selected candidate markers was analyzed and correlated with clinical parameters as well as short and long-term outcomes post LT. Whereas both, the calculated Eurotransplant Donor-Risk-Index and the donor body mass index, had either a poor or no predictive value concerning serum levels indicative for liver function (ALT, AST, GGT, bilirubin) after 6 months, chronological donor age was weakly predictive for serum bilirubin (AUC=0.67). In contrast, the major histcompatibility complex class I related chain A (MICA) mRNA expression demonstrated a high predictive value for serum liver function parameters revealing an inverse correlation (e.g. for ALT: 3 months p=0.0332; 6 months p=0.007, 12 months 0.0256, 24 months p=0.0098, 36 months, p=0.0153) and proved significant also in a multivariate regression model. Importantly, high expression of MICA mRNA revealed to be associated with prolonged graft survival (p=0.024; log rank test) after 10 years of observation, whereas low expression was associated with the occurrence of death in patients with transplant related mortality (p=0.031). Given the observed correlation with short and long-term graft function, we suggest MICA as a biomarker for pre-transplant graft evaluation.

## Introduction

Confronted with the growing gap between the number of patients on the wait list and the limited number of organs available for transplantation, transplant centres worldwide have steadily increased the utilization of livers from extended criteria donors (ECD). Whereas this practice constantly pushes the limits of what is acceptable for transplantation carrying the risk of inferior outcomes, some ECD grafts display excellent survival rates (1, 2). However, the identification of risk determinants for ECD organs is still complicated by the fact that the impact of seemingly obvious clinical parameters, such as chronological donor age, has been analysed in several studies with contradictory conclusions (3, 4). Therefore, it remains a challenge to define alternative approaches allowing the discrimination between ECD livers, which can be considered for transplantation and those needing to be excluded. In this regard, both the donor risk index (DRI) and in particular the Eurotransplant Donor-Risk-Index (ET-DRI) have been suggested as valuable instruments to score donor liver quality (5). Although useful, the validity of these scores remains limited in several facets (6). Consequently, the necessity persists to define alternative parameters as a metric for liver graft quality. In our previous studies on renal pre-implantation (zero-hour) biopsies, we identified a set of candidate genes to be characteristic of intragraft immune activation (7), and most importantly, especially the activating cytotoxicity receptor NKG2D appeared to be predictive for renal allograft function after 12 months post kidney transplantation (8). In this present study, aiming to investigate whether our earlier findings in kidney allografts may also be translated to LT, we analysed defined candidate genes in 88 liver zero-hour biopsies and evaluated their potential to predict allograft function up to 10 years post-transplantation. In addition to NKG2D, we focused on its ligand, the MHC class I poly-peptide-related sequence A (MICA), as well as markers indicating increased donor graft immunogenicity (HLA-DRB), immune activation (CCL19), obesity (Leptin) and alternative Natural Killer (NK) cell receptors (DNAM-1).

## Materials and Methods

### Subjects

We analysed 88 zero-hour biopsies from liver transplants conducted between February 2008 and December 2010 at the Medical University of Innsbruck, Austria. Specimens were retrieved from 1.5 cm^2^ subcapsular wedge biopsies taken from the left lobe during back-table preparation as described elsewhere (9). Afterwards, samples were immediately transferred to Allprotect Tissue Reagent (Qiagen, Hilden, Germany). Deceased donor LT was performed according to previously described techniques (10). Triple immunosuppression regimen was implemented as described earlier (10). The study was approved by the local Ethics Committee (UN5054, 324/4.13) and all experiments were conducted in compliance with the Declarations of Helsinki and Istanbul.

### Quantitative Real-Time RT-PCR

Total RNA was prepared as described (8, 11, 12). For cDNA synthesis, 1 µg of total RNA was incubated for 90 min at 42°C in a total volume of 20 µl containing 50 mM Tris-HCl pH 8.3, 50 mM KCl, 4 mM MgCl_2_, 10 mM DTT, 1 µM dT_18_ primer, 1 mM dNTPs, and 200 U of RevertAid H Minus M-MuLV Reverse Transcriptase (Thermo Scientific, Braunschweig, Germany). RT-PCR for gene expression analysis was performed applying the ABI PRISM 7500 Sequence Detection System (Life Technologies, Darmstadt, Germany) as we described recently (11). Primers for HPRT, NKG2D were designed using Primer Express Software (Life Technologies) and validated. Primers for HLA-DRB1, CC-chemokine ligand 19 (CCL19), Leptin, DNAX accessory molecule 1 (DNAM-1), and MICA were purchased as Assays on Demand (Life Technologies) (**Supplemental Table 1**).

### Clinical Variables

Clinical variables considered for analysis included recipient and donor age, donor type, recipient sex, BMI, cold ischemic time, warm ischemic time, donor and recipient infection status (CMV, HCV, HBV) as well as total bilirubin, alanine transaminase (ALT), gamma-glutamyl transpeptidase (GGT) and aspartate aminotransferase (AST). The ET-DRI was calculated as formerly defined by Braat et al. (5).

### Statistical Analysis

Analyses were performed using GraphPad Prism and the statistical software environment R (version 3.4.1) using packages survival, ROCR, car, dynpred, cmprsk. 95%-confidence interval of area under ROC curve was calculated using R package pROC based on a method by DeLong et al. (13). ROC curve (AUC) against random classification were calculated using R package verification and Mann-Whitney U (Wilcoxon) test.

Data were tested for normal distribution using the Kolmogorov-Smirnov test. To compare two groups, a two-sided Student’s T test, and to compare three groups, one-way ANOVA followed by Bonferroni posthoc tests were performed. Pearson product-moment correlation coefficient (and Spearman’s rank) with Fisher transformation was used to test the association between gene expression (correlation matrix) and the association between expression profiles and graft function. All variables were mean centered and scaled by standard deviation to make the coefficients of the individual regression models comparable (standardized coefficients). P values were adjusted for multiple hypothesis testing based on the false discovery rate according to the Benjamini-Hochberg method. Multi-collinearity between variables was excluded using the variance inflation factor. To test the ability of various markers to discern functional grafts from those with impaired function at 24 months post transplantation (with separate models for different functional outcome parameter according to respective cutoff for ALT >= 45 U/l, AST >=45 U/l, bilirubin >=1.2 mg/dl, and GGT >= 65 U/l indicating impaired function), univariable and multivariable logistic regression analyses were performed including MICA mRNA expression, donor age, BMI, and ET-DRI as independent variables. A leave-one-out cross-validation procedure (LOOCV) was performed to avoid overfitting. The AUC of the ROC was used as predictive value. Kaplan-Meier plots were used to analyze overall survival, and a log-rank test was applied to assess the statistical significance of differences between survival curves. Therefore, liver grafts were dichotomized at the maximal Harrel’s concordance index defining the cutoff for normalized expression data, whereby grafts with expression >cutoff were defined as high expression and ≤cutoff as low expression. Competing risks between transplantation related mortality (TRM) and other cause of deaths were analyzed by cumulative incidence curves and differences between grafts with low MICA expression versus high MICA expression using Gray test.

## Results

### Patient and Donor Characteristics

Of 88 investigated patients, 9 underwent re-transplantation. Mean recipient age was 54.9 ± 3.9 years and mean BMI was 25.1 ± 3.9. The calculated Model for End-stage Liver Disease (MELD) score for recipients was 15.7 ± 8.1 and in total, 24 patients were diagnosed with a hepatocellular carcinoma. All grafts were derived from brain dead (DBD) donors. Donor mean age was 50.1 ± 16.5 years and the calculated ET-DRI was 1.7 ± 0.4. Overall, 21 patients experienced an episode of acute cellular rejection within the first-year post-transplantation (**Table 1**).

**Table 1:** Demographic data of liver donors and recipients.

Patient characteristics	n (%)	Mean	SD	Range
**Gender (male/female)**	64 (72.7)/24(27.3)			
**Age (yr)**	88 (100.0)	54.9	3.9	54.9
**Weight (kg)**	88 (100.0)	76.0	15.0	65.0
**BMI (kg/m^2^)**	88 (100.0)	25.1	3.9	16.9
**labMELD**	88 (100.0)	15.7	8.1	35
**Cause of ESLD**				
** ALD**	29 (33.0)			
** Viral hepatitis**	27 (30.7)			
** NASH**	11 (12.5)			
** cryptogenic**	3 (3.4)			
** PSC**	3 (3.4)			
** PBC**	4 (4.5)			
** Other**	11 (12.5)			
** HCC**	24 (27.3)			
**Donor characteristics**				
**Gender (male/female)**	43 (48.9)/45 (51.1)			
**Age (yr)**	88 (100.0)	50.1	16.5	65.0
**GGT (U/l)**	87 (98.9)	101.8	145.7	669.0
**Donation after cardiac death**	0 (0.0)			
**Rescue allocation**	3 (3.4)			
**Donor procurement**				
** Local**	26 (29.5)			
** Regional**	45 (51.1)			
** Extra-regional**	17 (19.3)			
**Cause of death**				
** Trauma**	16 (18.2)			
** Anoxia**	7 (8.0)			
** CVA**	63 (71.6)			
** Other**	2 (2.3)			
** ET-DRI**	88 (100.0)	1.7	0.4	1.5
**Transplant characteristics**				
**Technique**				
** Cava-replacing**	87 (98.9)			
** Piggy back**	1 (1.1)			
**Previous transplants**	9 (10.2)			
**High-urgency transplant**	3 (3.4)			
**Cold ischemia period (hr)**	88 (100.0)	8.9	2.8	16.0
**Warm ischemia period (min)**	88 (100.0)	41.7	9.6	54.0
**Operation time (min)**	87 (98.9)	366.7	112.7	585
**Split graft**	0 (0.0)			
**Acute rejection within first year (yes/no)**	21 (23.9)/67(76.1)			

CVA, cerebrovascular accident; ESLD, end-stage liver disease; ALD, alcoholic liver disease; NASH, nonalcoholic fatty liver disease; PSC; primary sclerosing cholangitis; PBC, primary biliary cholangitis.

### Livers Derived From Donors With a BMI >30 Display an Inflammatory Profile

**Figure 1:**
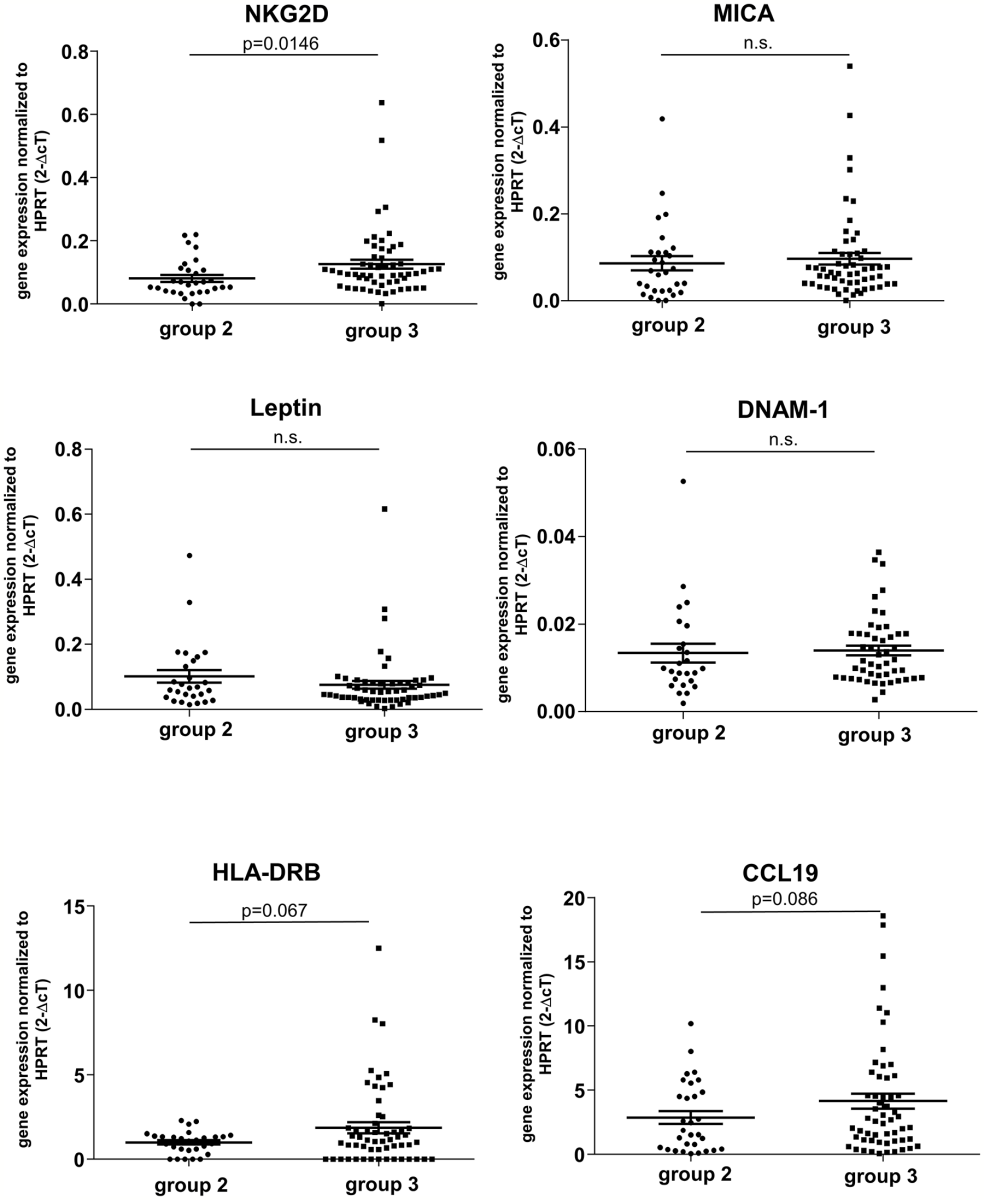
mRNA expression of candidate genes in liver zero-hour biopsies according to donor BMI. Donor groups were defined as summarized in **Supplemental Table 2**. Gene expression was measured by real-time RT-PCR for the cytotoxicity receptor NKG2D and its ligand MICA, the adipokine leptin, the activating receptor DNAM-1, HLA-DRB and the chemokine CCL19. With the exception NKG2D all markers investigated did not demonstrate an elevated gene expression level in liver grafts derived from overweight and obese donors (Group 3, BMI: 25-29.9 kg/m²) compared with normal weight donors (Group 2, BMI: 18.5-24.9 kg/m²). Data of evaluable values are presented as mean values ± SEM. Statistically significant differences between normal and overweight donors were tested with two-sided Student’s T test. n.s., not significant.

Since the influence of obesity on overall survival post-LT is controversially discussed (14, 15), we first focused on donor BMI as a potential risk factor to impact liver graft quality. Recently, we illustrated that the natural cytotoxicity receptor NKG2D is a marker of renal senescence and correlates with allograft function (8). Interestingly, by classifying 84 liver grafts according to a BMI standard categorization (16) (normal weight: 18.5-24.9 kg/m², Group 2, n=31 and overweight/obese: 25-29.9 kg/m², Group 3, n=57) (**Supplemental Table 2**), we detected a significant induction of NKG2D mRNA in specimens defined as Group 3 compared with Group 2 (p=0.0146) (**Figure 1**). In contrast, the ligand of NKG2D called MICA, did not significantly differ between both groups. We further examined Leptin, an adipokine, which has been associated with obesity (17, 18), but could not identify significant changes between both investigated BMI groups. In order to confirm an increase of NK cell receptors, we studied DNAM-1 (also known as CD226) (19). Analogous to Leptin, no differences were found between both BMI groups. In addition, we measured the expression of the MHC class II molecule HLA-DRB, as an indicator for increased immunogenicity in the graft as well as CCL19. Although we detected an mRNA increase for both markers in Group 3 versus Group 2, this difference was not statistically significant (**Figure 1**).

### Donor Livers With an Advanced Age >55 Years Display a Higher mRNA Expression of the NKG2D Ligand MICA

According to our previous observations that kidney organs >55 years are characterized by an inflammatory profile (8), we aimed to identify whether this is also applicable in liver grafts. In analogy to our previous approach (8), working with a large number of available specimens allowed to subdivide into three groups according to donor age, classified as ≤30 years (n=14, Group 1 or young), 31-54 years (n=36, Group 2 or middle-aged) and ≥55 years (n=38, Group 3 or old). In contrast to previous observations in the kidney, we could not detect elevated mRNA expression levels of NKG2D in livers at advanced age. In addition, no induced mRNA levels were observed for Leptin, DNAM-1 and HLA-DRB. However, MICA mRNA was significantly elevated in Group 3 compared to Group 1 (p=0.035) and a similar expression profile was detected for CCL19 (p=0.015) (**Supplemental Figure 1**).

### CCL19 Correlates With Donor BMI or Donor Age

**Figure 2:**
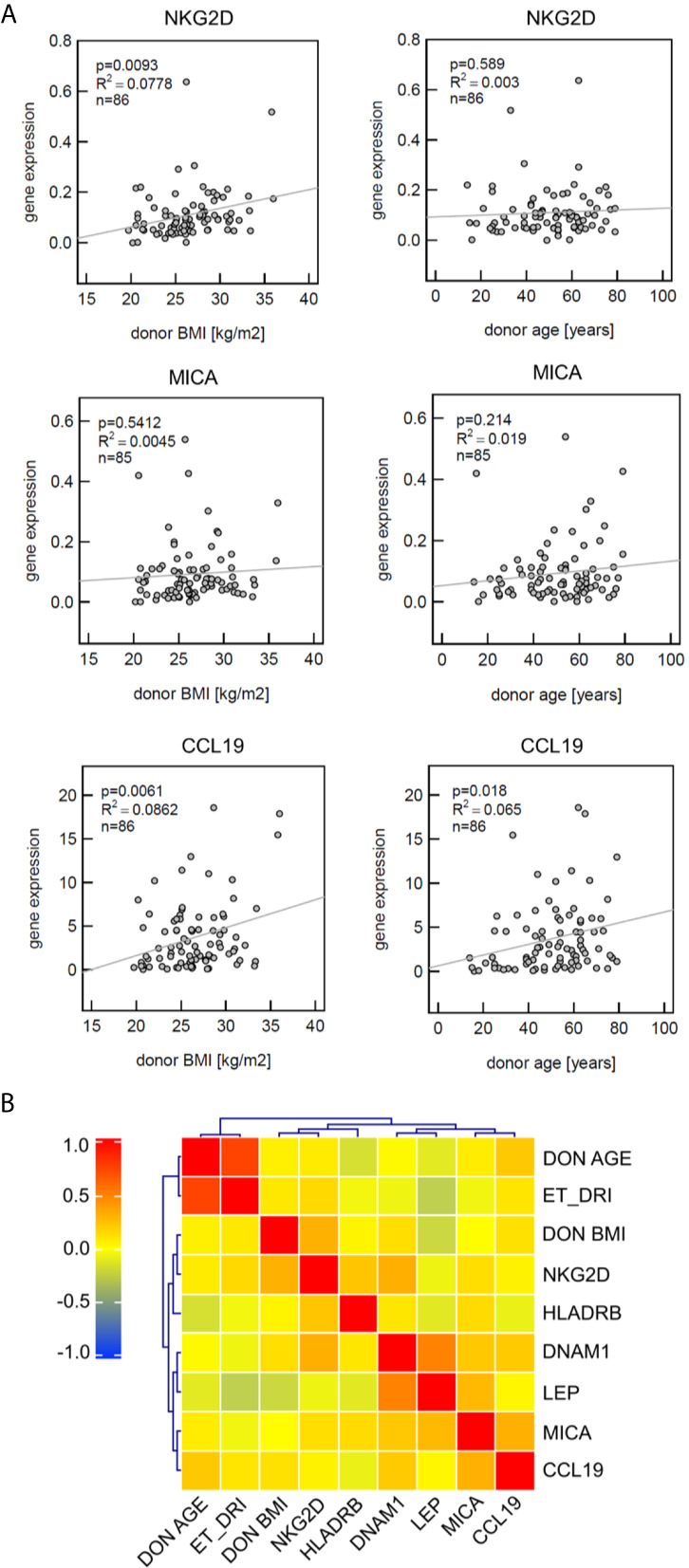
Analysis of defined markers for correlation with clinical parameters and coexpression profile. **(A)** Gene expression of CCL19 significantly correlates with both donor BMI and donor age, NKG2D with donor BMI. Linear regression analysis was performed; the coefficient of determination (R^2^) indicating the goodness-of-fit and the p-values are provided. **(B)** Heat map visualizing the correlation matrix of gene expression levels in liver zero-hour biopsies. MICA expression patterns are not associated with donor age or donor BMI. Spearman’s rank correlation coefficient is presented according to the color scheme on the right. Hierarchical clustering was used to group genes with similar profiles as well as the risk factors donor BMI, donor age and Eurotransplant donor risk index (ET-DRI).

Linear regression analysis demonstrated that NKG2D significantly correlates with donor BMI but not with age. The strongest positive correlation with both risk factors was observed for CCL19, whereas MICA mRNA expression neither correlated with BMI nor age (**Figure 2A**).

This was confirmed by use of hierarchical cluster analyses (Heat map), visualizing the correlation matrix of gene expression levels in liver zero-hour biopsies. This approach confirmed that MICA expression patterns are not associated with donor age or BMI, whereas MICA and CCL19 showed a co-clustering in this analysis (**Figure 2B**).

### MICA mRNA Expression Is Predictive for Liver Function Post-Transplantation

**Figure 3:**
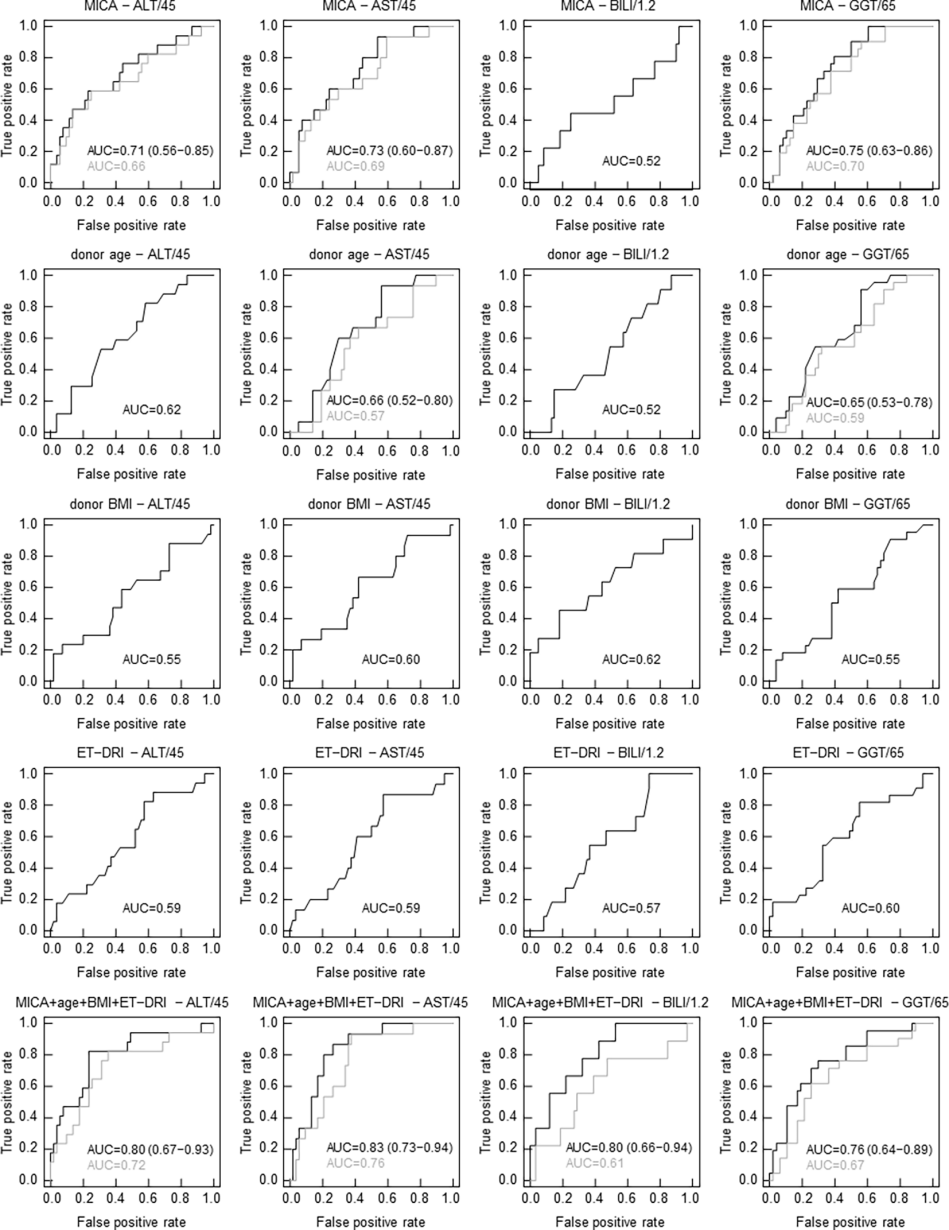
In contrast to clinical parameters, MICA mRNA expression correlates with graft function 24 months following LT. Receiver operating characteristics (ROC) curves from univariate and multivariate logistic regression (LR) analyses to discern functional livers from those with impaired function (based on a cutoff for ALT of 45 U/l, for AST of 45 U/l, for bilirubin of 1.2 mg/dl and for GGT of 65 U/l in serum 24 months post-transplantation). ROC curves showing true-positive rate (sensitivity) versus false-positive rate (1-specificity) for individual LR classifiers based on MICA mRNA expression, donor age, donor BMI, ET-DRI and combined multivariate LR classifiers. Results from leave-one-out cross-validation (LOOCV) are indicated in gray. The area under the ROC curve (AUC) serves as a predictive value. (LOOCV was only evaluated and shown when AUC ≥ 0.65 including all grafts).

To test a potential predictive value of donor age, BMI, ET-DRI, as well as the expression of the most promising candidate, MICA, for graft function post-transplantation, logistic regression analysis was performed. As evident in **Figure 3**, MICA revealed to be predictive for liver function after 24 months, with an AUC of 0.71 (95%CI: 0.56-0.85, p=0.0057, AUCLOOCV=0.66) for ALT and 0.73 (95%CI: 0.60-0.87, p=0.0031, AUCLOOCV=0.69) for AST. This predictive value is superior compared that of the chronological donor age (AUC of 0.62). Combined multivariable logistic regression analysis including MICA mRNA expression, donor age, BMI, and ET-DRI as independent variables revealed a predictive value for liver function 24 months post-transplantation with an AUC of 0.80 (95%CI: 0.67-0.93, p<0.001, AUCLOOCV=0.72) for ALT and 0.83 (95%CI: 0.73-0.94, p<0.001, AUCLOOCV=0.76) for AST. Applying Spearman rank correlation analysis in order to correlate mRNA expression with functional liver data 0-36 months post-transplantation, especially MICA showed to be associated with clinical parameters, revealing an inverse correlation for the parameters ALT, AST and GGT and bilirubin (e.g. for ALT: 3 months p=0.0332; 6 months p=0.007, 12 months 0.0256, 24 months p=0.0098, 36 months, p=0.0153, **Table 2** and **Supplemental Table 3**). Importantly, neither NKG2D nor CCL19 showed comparable results (**Supplemental Table 4**).

**Table 2:** Univariate linear regression and correlation analysis depicting the predictive power of MICA post liver transplantation.

Gene	functional parameter	n	Stand. coeff.	Linear regression			Adj R^2^	Spearman rank correlation		
				*P*	Adj *P*	R^2^		ρ	*P*	Adj *P*
**MICA**	0 Mo AST	83	-0.081	0.4676	0.5698	0.007	-0.006	-0.224	**0.0416**	0.2121
**MICA**	0 Mo ALT	83	-0.111	0.3160	0.6483	0.012	0.000	-0.212	0.0545	0.3271
**MICA**	3 Mo AST	78	-0.236	**0.0372**	0.2232	0.056	0.043	-0.312	**0.0054**	**0.0327**
**MICA**	3 Mo ALT	78	-0.300	**0.0077**	**0.0463**	0.090	0.078	-0.311	**0.0055**	**0.0332**
**MICA**	3 Mo GGT	78	-0.284	**0.0117**	**0.0705**	0.081	0.069	-0.303	**0.0070**	**0.0421**
**MICA**	6 Mo BILI	73	-0.056	0.6384	0.8766	0.003	-0.011	-0.049	0.6827	0.7820
**MICA**	6 Mo AST	73	-0.265	**0.0233**	0.1397	0.070	0.057	-0.221	0.0603	0.3615
**MICA**	6 Mo ALT	73	-0.442	**0.0001**	**0.0005**	0.195	0.184	-0.437	**0.0001**	**0.0007**
**MICA**	6 Mo GGT	73	-0.248	**0.0344**	0.2063	0.062	0.048	-0.188	0.1120	0.6721
**MICA**	12 Mo AST	76	-0.183	0.1140	0.6837	0.033	0.020	-0.304	**0.0076**	**0.0454**
**MICA**	12 Mo ALT	76	-0.260	**0.0232**	0.1390	0.068	0.055	-0.324	**0.0043**	**0.0256**
**MICA**	12 Mo GGT	76	-0.201	0.0819	0.4916	0.040	0.027	-0.299	**0.0087**	**0.0522**
**MICA**	24 Mo AST	69	-0.272	**0.0238**	0.0715	0.074	0.060	-0.398	**0.0007**	**0.0043**
**MICA**	24 Mo ALT	69	-0.348	**0.0034**	**0.0204**	0.121	0.108	-0.372	**0.0016**	**0.0098**
**MICA**	24 Mo GGT	69	-0.219	0.0705	0.3383	0.048	0.034	-0.295	**0.0140**	0.0837
**MICA**	36 Mo AST	67	-0.206	0.0946	0.3565	0.042	0.028	-0.279	**0.0221**	0.1325
**MICA**	36 Mo ALT	67	-0.295	**0.0153**	0.0919	0.087	0.073	-0.334	**0.0057**	**0.0340**

Adj P, adjusted P value based on the false discovery rate according to the Benjamini-Hochberg method considering all analyzed genes; Adj R2, coefficient of determination (adjusted R2) multivariate R; d graft function; AST, aspartate aminotransferase; ALT, alanine transaminase; GGT, gamma-glutamyl transpeptidase; Bili, Bilirubin at discharge, 3, 6 12, 24 and 36 months after transplantation; Stand coeff, standardized coefficient in the respective regression model. P values and adjusted P values < 0.05 are in bold.

### Only MICA mRNA Expression Is Predictive for Liver Function Post-Transplantation in a Multivariate Analysis

In order to further delineate the predictive value of MICA for graft function, a multivariate logistic regression analysis was performed. In contrast to all other analyzed parameters, only MICA mRNA expression remained a significant variable in the multivariate regression model (**Supplemental Table 5**).

### High MICA Expression Is Predictive for Improved Graft Survival But Not for Acute Rejection Episodes

**Figure 4:**
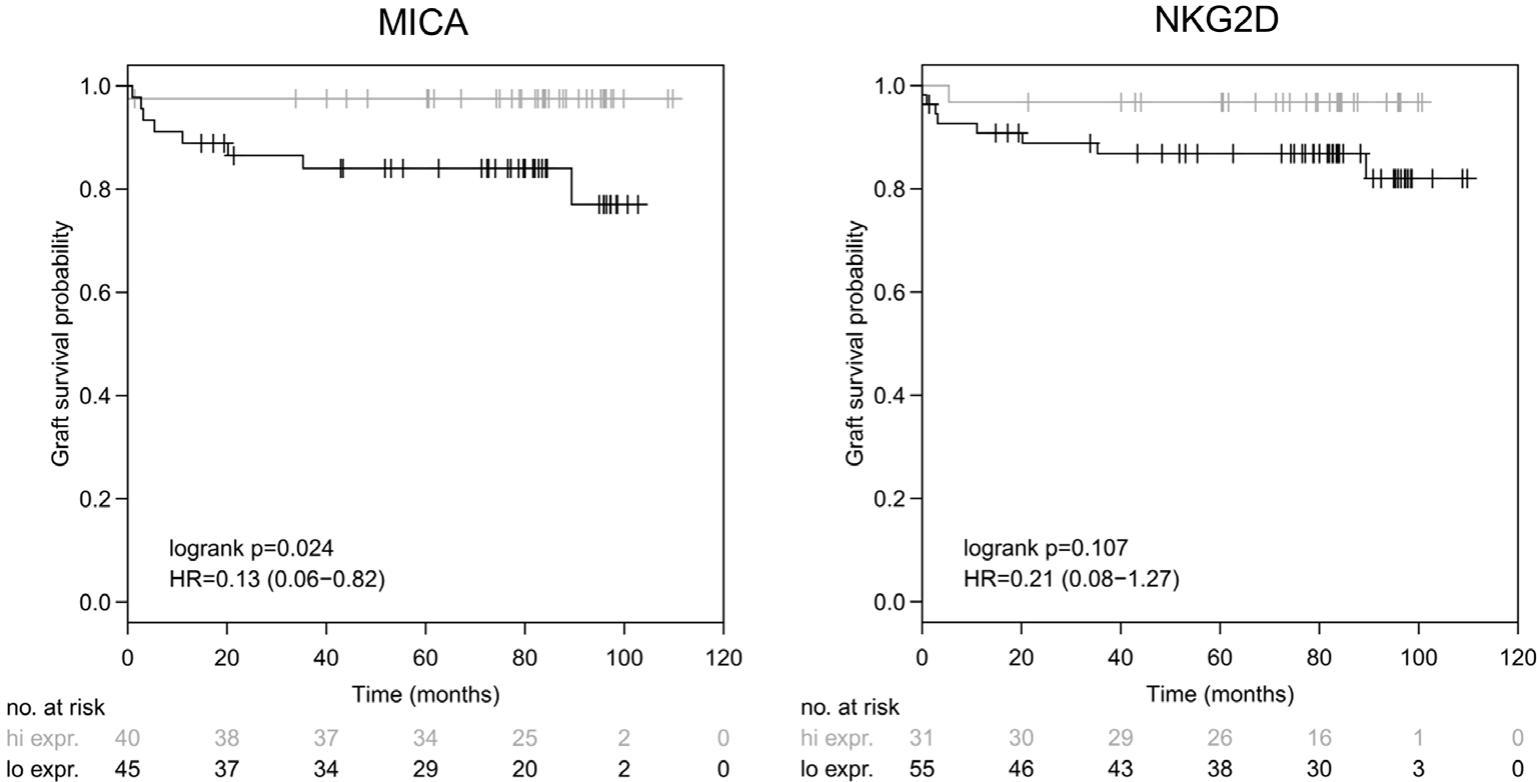
Intragraft mRNA expression of MICA correlates with long-term survival. MICA mRNA illustrates a significant correlation with graft survival following LT (logrank p = 0.024) after 10 years of follow-up [left], whereas NKG2D showed no significant correlation with graft survival (logrank p=0.107) [right]. Dichotomization cutoff for normalized expression was defined at the maximal Harrel’s concordance index (MICA, C_max_ = 0.68, cutoff = 0.071 and NKG2D, C_max_ = 0.63, cutoff = 0.1081), whereby high expression was defined > cutoff and low expression ≤ cutoff.

Having observed that MICA expression is indicative for liver function, next, we addressed whether MICA does also have a predictive value for graft survival. Indeed, in contrast to all other parameters (including ET-DRI), high intragraft expression of MICA showed a significant correlation with long-term graft survival after 10 years of follow-up (logrank p=0.024). To differentiate between high and low MICA expression, a dichotomization cutoff for normalized expression was defined at the maximal Harrel’s concordance index (MICA, C_max_=0.68, cutoff=0.071), whereby high expression was defined >cutoff and low expression ≤cutoff. In contrast, this was not the case for any other of the applied variables including NKG2D (logrank p=0.107; C_max_=0.63, cutoff=0.1081), DNAM-1 (logrank p=0.528), HLA DRB (logrank p=0.804), leptin (logrank p=0.758) and CCL19 (logrank p=0.860) (data not shown, **Figure 4**). Finally, we tested for a possible association between MICA expression and the occurrence of acute rejection episodes within the first year. However, logistic regression analysis revealed no significant correlation (p=0.096; AUC=0.65, data not shown).

### Low MICA Expression Is Associated With Patient Death

**Figure 5:**
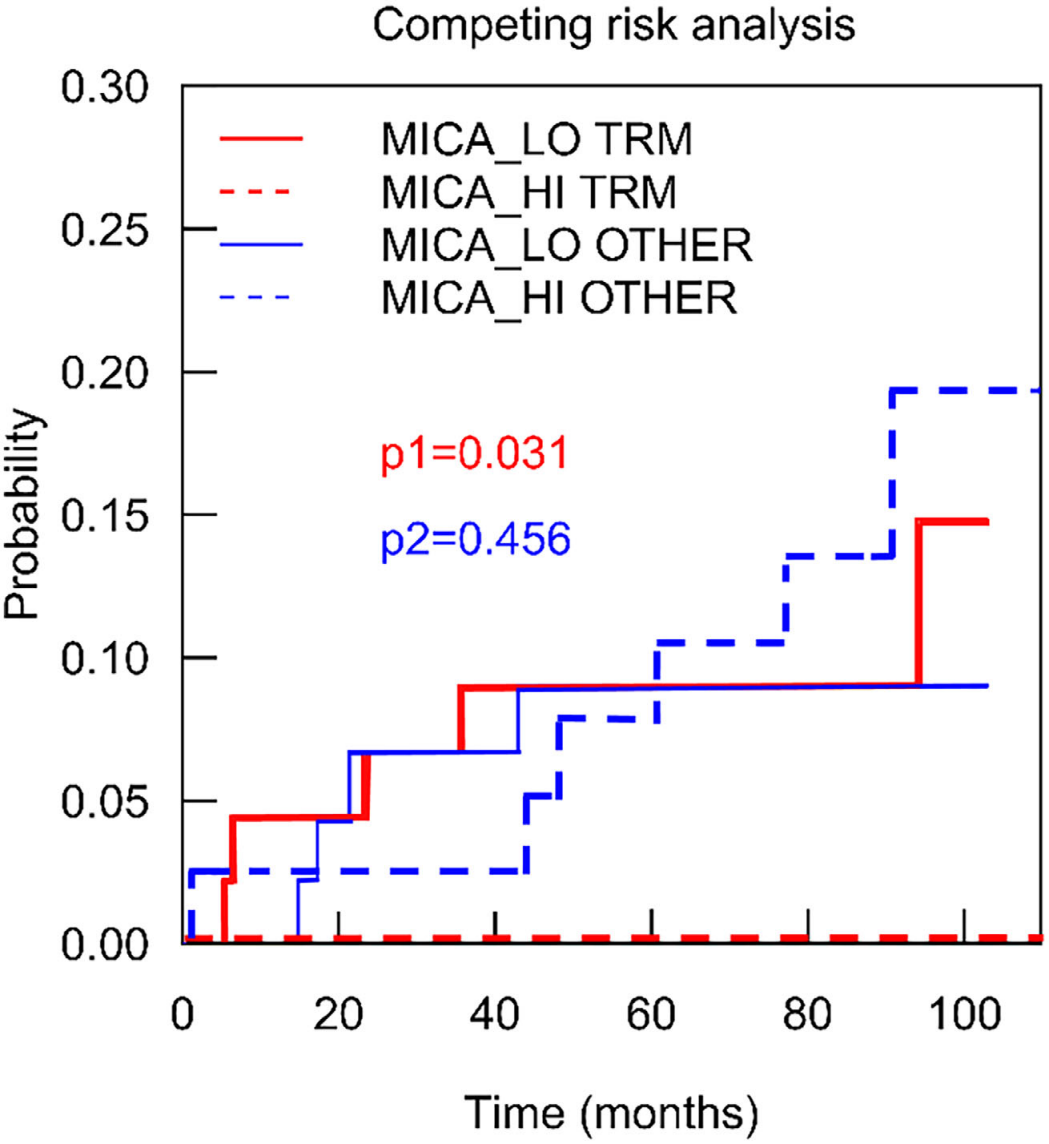
Intragraft mRNA expression of MICA correlates transplant related mortality. To depict the association of MICA expression on mortality in detail, a competing risk analysis was calculated analyzing patients with transplant related mortality (red) and such patients with other causes of death (death with functioning graft) (blue). Low MICA expression (red line) was significantly associated with the occurrence of death in TRM patients (p = 0.031, Gray Test).

In order to further delineate the impact of MICA expression on patient death, cumulative incidence functions were analysed in a competing risk analysis which was calculated differentiating between patients with TRM and patients with other causes of death (death with functioning graft). As shown in **Figure 5**, compared to high MICA expression, low MICA expression was significantly associated with the occurrence of death in TRM patients (p=0.031, Gray Test). On the other hand, in such patients with other causes of death, MICA expression did not significantly differ (p=0.456). These data underline the validity of MICA as a predictive marker for LT outcomes.

## Discussion

To compensate the immanently high wait list mortality, the transplant community is turning to the utilization of marginal and high-risk grafts (1, 20–22). The term “marginal” refers to a not well-defined group of donors with characteristics such as advanced age, displaying steatosis, hepatitis C virus positive serology or donation after circulatory death (DCD) (22). However, these descriptive attributes alone remain insufficient to predict the specific risk of transplanting an individual graft. The analysis of zero-hour biopsies may therefore provide additional information about these organs. Indeed, pre-transplant biopsy retrieval has become a widely accepted standard in many centers worldwide. However, so far, such specimens are mainly used to evaluate the grade of steatosis/fibrosis by microscopic frozen section histology (9).

In a recent study, we illustrated that the potential scope of zero-hour biopsy analysis can by far exceed the information obtained from histology in kidney allografts as we could show that the classical histo-morphologic assessment (Remuzzi biopsy score) could predict allograft outcome, although the predictive value of molecular markers (NKG2D) proved to be significantly superior (8). In this present study, we translated these findings derived from kidney allografts to investigate whether a defined set of inflammatory genes may also be applicable to predict outcomes after LT. In Austria, organs for transplantation are mainly procured from BD donors (23), a circumstance that allowed us to exclusively include BD graft samples and avoid potential biasing effects from a heterogenous BD/DCD study group.

Our analysis demonstrates that intragraft expression of NKG2D, an activating receptor expressed on NK cells and which has also been identified on γδ T, CD8^+^ αβ T, and NKT lymphocytes (24–26), was significantly induced in livers derived from donors with a BMI 25-29.9 kg/m² (**Figure 1**). Interestingly, in strong contrast to our prior findings in kidney allografts (8), NKG2D expression in liver grafts was not significantly affected by donor age (**Supplemental Figure 1**). Steatotic livers represent the most common type of “marginal” organs that have been introduced during the last two decades. A precise definition and reproducible method for steatosis quantification is currently solely based on the histological assessment. We therefore analysed the expression of leptin, since leptin has been reported to augment both inflammatory and profibrogenic responses in the liver and plays a role in the pathogenesis of hepatic steatosis (27, 28). However, we were not able to detect an increase of leptin mRNA expression in livers from donors with a BMI 25-29.9 kg/m², nor could we detect an induction in livers with an advanced age. Contrary to a prior study reporting on a possible role of leptin as a biomarker in kidney transplantation (29), our study indicates that leptin has no predictive value in the setting of LT.

Likewise, the expression of the markers CCL19, DNAM1 and HLA-DRB, all of which were chosen due to their relevance in kidney transplantation (7, 8), did not show a significant predictive value for liver graft survival or function.

In contrast, the most promising candidate turned out to be the NGK2D ligand, the non-HLA antigen MICA. Since MICA, unlike the classical HLA molecules, is not involved in antigen presentation to T cells but *via* NKG2D recognition interacts with human NK cells, γδ T, mucosal-associated invariant T (MAIT), CD56⁺ T, and CD8⁺ T cells (30), MICA is unique to the extent that it plays a key role in linking the innate and adaptive immune responses in organ transplantation (31). This circumstance has drawn considerable attention in the scientific community, and the role of this non-HLA antigenic target has been extensively studied in the setting of kidney transplantation (31). In contrast, as outlined by recent review articles, the role of MICA in LT currently remains unclear due to a lack of available data (31, 32). To our knowledge, only two prior studies exist assessing the potential influence of MICA on LT outcomes (33, 34). Both investigated anti-MICA antibodies in patients’ sera and only one study investigated MICA expression profiles in liver graft samples. The authors state that they found only weak mRNA levels for MICA in liver cells but no protein or cell surface expression (34). However, in contrast to this study, we used whole liver tissue lysates for a comprehensive mRNA analysis. With this approach, we found a significant correlation of MICA expression with allograft function after 3, 6, 12, 24 and 36 months, which was confirmed in the multivariate analysis. MICA expression revealed to be independent of donor age or BMI, but high expression of MICA revealed to be an independent predictor of long-term allograft survival after 10 years post-transplantation. Importantly, low MICA expression was significantly associated with the occurrence of patient death.

Noteworthy, our observation that an elevated mRNA expression of MICA is associated with improved outcomes after LT seems paradoxical at a first glance. Since MICA represents a ligand for NK and T cell receptors, it could be expected such cells displaying high MICA expression could be rendered for killing by cytotoxic lymphocytes. This mechanism was proven in various tumor cell lines which strongly expressed MICA on the cell surface. However, in a wide range of normal epithelia, MICA was shown to be broadly expressed, but only in distinct intracellular structures with only occasional (<20%) membrane localization in a few normal tissue types (bladder, bronchus, kidney and colon) (35). Accordingly, our experiments show that MICA was expressed in liver tissue samples on an mRNA level, whereas no MICA expression was found on the cell surface. This finding could serve as an explanation why anti-MICA antibodies in the recipient seem to have no clinical significance. Although we did not assess patient sera for anti-MICA antibodies in our patients, the studies by Ciszek et al. and Uzunel et al. (33, 34) report that, against initial expectations, the presence of anti-MICA antibodies in patients’ sera were not associated with deteriorated outcomes.

In response to cellular stress, MICA expression is induced in many cell types, including epithelium or fibroblasts, whereas MICA expression on hepatocytes or Kupffer cells of has not been demonstrated so far. However, it has been shown that levels of MICA/B mRNA positively correlate with the stage of fibrosis, suggesting that MICA/B also contribute to the progression of liver fibrosis (36). In addition, MICA expressing T cells have been shown to be enriched within HBV-infected livers compared with the periphery or to healthy livers (37). Both observations clearly demonstrate that MICA can be up-regulated as a consequence of inflammatory pathology, independent of the cell type. Thus, our results suggest that the detection of MICA mRNA in liver zero-hour biopsies detects sub-clinical changes in the graft, which are predictive for outcomes, but beyond the scope of classical histopathological evaluation.

One possible limitation to this study is that we propose a biomarker which requires several hours for its assessment (e.g. *via* RT-PCR). In consideration of the immanent time pressure to minimize cold ischemia storage time one might argue that such a marker is simply not feasible for solid organ transplantation. However, regarding the rapid technological advances in ex situ organ preservation, such concerns might soon be a matter of the past. Both, cold (38, 39) as well as warm (40, 41) liver perfusion systems are now able to significantly extend preservation times. Consequently, ECD livers which would have formerly been discarded can now be considered for transplantation after the assessment of graft quality. Therefore, new biomarkers are urgently needed to predict liver function after subsequent transplantation (38). As recently formulated by Verhoeven *et al.*, ideally, such biomarkers could help to enlarge the donor pool by objectively screening liver grafts that initially would be discarded based on their predisposing characteristics (42).

Based on our findings illustrating that MICA gene expression in zero-hour liver biopsies correlates with both, short and long-term graft function, we suggest MICA as a candidate biomarker for future pre-transplant graft evaluation.

## Data Availability Statement

The original contributions presented in the study are included in the article/**Supplementary Material**, further inquiries can be directed to the corresponding authors.

## Ethics Statement

The studies involving human participants were reviewed and approved by the Ethics Committee of the Medical University Innsbruck, Innsbruck, Austria. The patients/participants provided their written informed consent to participate in this study.

## Author Contributions

TR and KK were responsible for the conception of the project, literature review, data analysis, interpretation of results and write up of the manuscript. HH performed the statistical analyses. HE, JG, HS, PR, SE, MM, VM, MB, RÖ, HZ, and SS contributed to the acquisition of data, data analysis and interpretation of results. All authors contributed to the article and approved the submitted version.

## Funding

This study was supported by a grant from the Deutsche Forschungsgemeinschaft to KK (Ko-2270/4-1).

## Conflict of Interest

The authors declare that the research was conducted in the absence of any commercial or financial relationships that could be construed as a potential conflict of interest.
