# Supplementary material for: Expression of MICA in Zero Hour Biopsies Predicts Graft Survival After Liver Transplantation
Source: Front Immunol. 2021 Jul 20;12:606146. doi: 10.3389/fimmu.2021.606146 (PMC8329650; doi:10.3389/fimmu.2021.606146)
Supplement: Supplementary file 1 [file DataSheet_1.docx]

Supplementary Material

**Content:**

**Supplemental Figure 1. mRNA expression of candidate genes in liver zero-hour biopsies according to donor age.**

**Supplemental Table 1: Primer sequences.**

**Supplemental Table 2. Donor BMI groups.**

**Supplemental Table 3.** **Univariate linear regression analysis of candidate markers.**

**Supplemental Table 4. Univariate linear regression and correlation analysis depicting the predictive power of CCL19 and NKG2D post liver transplantation.**

**Supplemental Table 5. Multivariate linear regression analysis of candidate markers.**

**
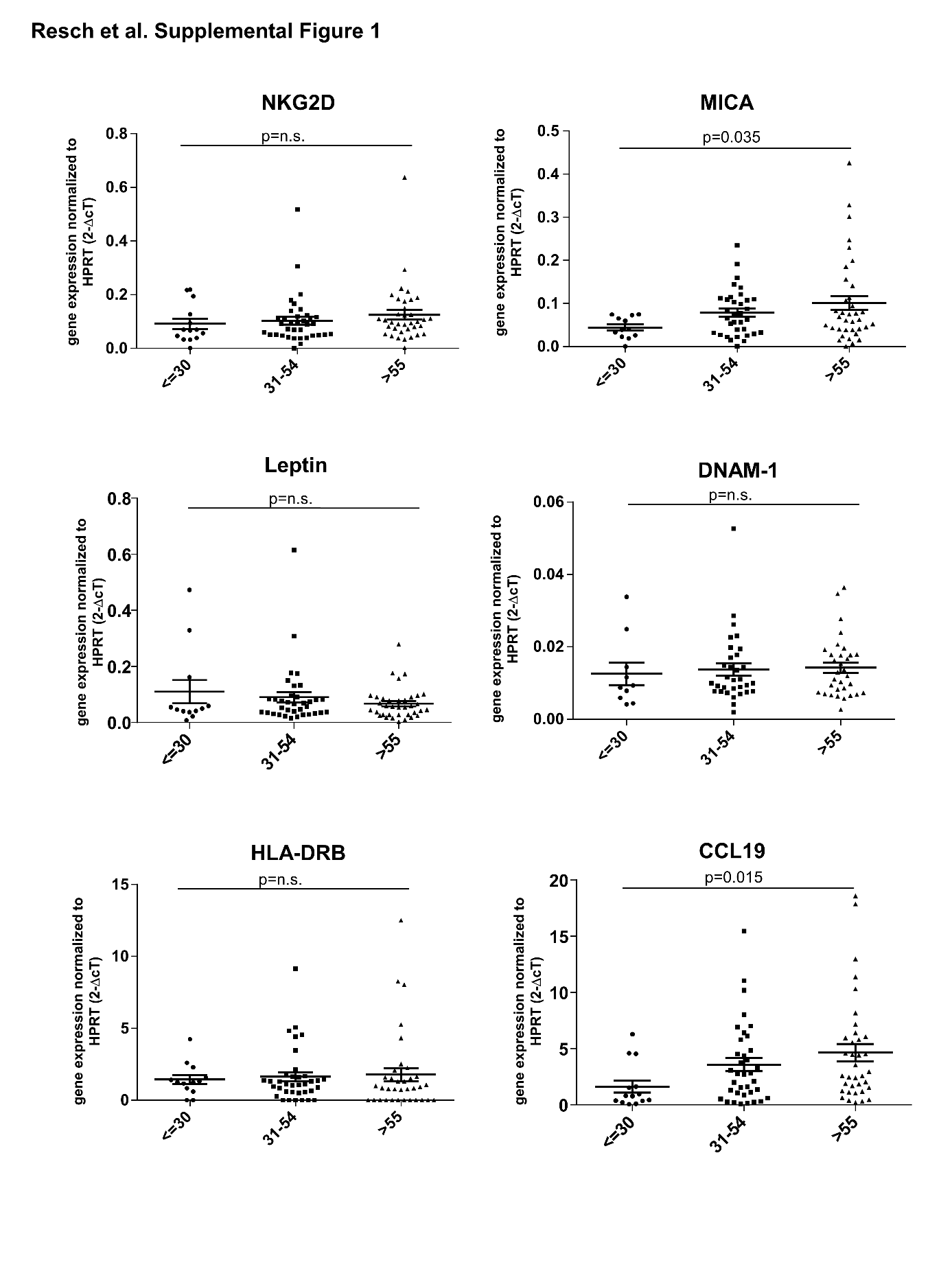
**

**Supplemental Figure 1. mRNA expression of candidate genes in liver zero-hour biopsies according to donor age.** Donor age groups were defined as ≤30 years (n=14, Group 1 or young), 31-54 years (n=36, Group 2 or middle-aged) and ≥55 years (n=38, Group 3 or old). A significant gene induction was detected for CCL19 and MICA in Group 3 versus Group 1, whereas the additional investigated markers did not illustrate an induced induction according to enhanced age. Data of evaluable values are presented as mean values ± SEM. Statistically significant differences between aged and young donors were tested with one-way analysis of variance (ANOVA) and Bonferroni post-hoc tests.

**Supplemental Table 1: Primer sequences.**

| Gene of interest | sense (5'-3') | anti-sense (3'-5') | probe (5'FAM - TAMRA3') |
| --- | --- | --- | --- |
| NKG2D | GGC TCC ATT CTC TCA CCC A | TAA AGC TCG AGG CAT AGA GTG C | CCT ACT AAC AAT AAT TGA AAT GCA GAA GGG AGA CTG |
| HPRT | AGT CTG GCT TAT ATC CAA CAC TTC G | GAC TTT GCT TTC CTT GGT CAG G | TTT CAC CAG CAA GCT TGC GAC CTT GA |
|  |  |  |  |
|  |  |  |  |
| Assay on Demand | **Assay ID** |  |  |
| DRB1 | HS00734212_m1 |  |  |
| CCL19 | HS00171149_m1 |  |  |
| Leptin | HS00174877_m1 |  |  |
| DNAM-1 | HS00170832_m1 |  |  |
| MICA | HS00792195_m1 |  |  |

| Donor BMI Group | n | mean | SEM | min | max |
| --- | --- | --- | --- | --- | --- |
| All | 88 | 26.38 | 3.64 | 19.72 | 36 |
| 1 (underweight:  BMI: <18.5 kg/m^2^) | 0 | - | - | - | - |
| 2 (normal weight:  BMI: 18.5 - 24.9 kg/m²) | 31 | 22.77 | 1.67 | 19.72 | 24.98 |
| 3 (overweight and obese:  BMI: 25 – 30+ kg/m²) | 57 | 28.44 | 2.66 | 25.06 | 36 |
| overweight  BMI: 25 - 29.9 kg/m² | 43 | 27.19 | 1.42 | 25.06 | 29.8 |
| obese  BMI: 30+ kg/m² | 14 | 32.26 | 1.78 | 30.35 | 36 |

**Supplemental Table 2. Donor BMI groups.**

**Supplemental Table 3.** **Univariate linear regression analysis of markers.** Adj P, adjusted P value based on the false discovery rate according to the Benjamini-Hochberg method considering all analyzed genes; Adj R2, coefficient of determination (adjusted R2) multivariate R; d graft function; AST, aspartate aminotransferase; ALT, alanine transaminase; GGT, gamma-glutamyl transpeptidase; Bili, Bilirubin at discharge, 3, 6 12, 24 and 36 months after transplantation; Stand coeff, standardized coefficient in the respective regression model. Adjusted P values < 0.05 are in bold.

| Gene | Outcome | n | Stand coeff | Adj R^2^ | Adj *P* |
| --- | --- | --- | --- | --- | --- |
| NKG2D | 0 Mo ALT | 84 | 0.087 | -0.005 | 0.6483 |
| HLADRB | 0 Mo ALT | 83 | 0.127 | 0.004 | 0.6483 |
| DNAM1 | 0 Mo ALT | 76 | -0.049 | -0.011 | 0.8119 |
| MICA | 0 Mo ALT | 83 | -0.111 | 0.000 | 0.6483 |
| Leptin | 0 Mo ALT | 81 | -0.009 | -0.013 | 0.9376 |
| CCL19 | 0 Mo ALT | 84 | 0.108 | 0.000 | 0.6483 |
| NKG2D | 3 Mo ALT | 79 | -0.037 | -0.012 | 0.8402 |
| HLADRB | 3 Mo ALT | 78 | -0.054 | -0.010 | 0.8402 |
| DNAM1 | 3 Mo ALT | 72 | 0.083 | -0.007 | 0.8402 |
| MICA | 3 Mo ALT | 78 | -0.300 | 0.078 | **0.0463** |
| Leptin | 3 Mo ALT | 76 | -0.024 | -0.013 | 0.8402 |
| CCL19 | 3 Mo ALT | 79 | 0.099 | -0.003 | 0.8402 |
| NKG2D | 6 Mo ALT | 74 | -0.215 | 0.033 | 0.1977 |
| HLADRB | 6 Mo ALT | 73 | -0.177 | 0.018 | 0.2695 |
| DNAM1 | 6 Mo ALT | 68 | -0.089 | -0.007 | 0.7086 |
| MICA | 6 Mo ALT | 73 | -0.442 | 0.184 | **0.0005** |
| Leptin | 6 Mo ALT | 71 | -0.020 | -0.014 | 0.8675 |
| CCL19 | 6 Mo ALT | 74 | -0.035 | -0.013 | 0.8675 |
| NKG2D | 12 Mo ALT | 77 | -0.031 | -0.012 | 0.9506 |
| HLADRB | 12 Mo ALT | 76 | -0.048 | -0.011 | 0.9506 |
| DNAM1 | 12 Mo ALT | 71 | 0.094 | -0.005 | 0.8681 |
| MICA | 12 Mo ALT | 76 | -0.260 | 0.055 | 0.1390 |
| Leptin | 12 Mo ALT | 74 | 0.111 | -0.001 | 0.8681 |
| CCL19 | 12 Mo ALT | 77 | -0.007 | -0.013 | 0.9535 |
| NKG2D | 24 Mo ALT | 70 | -0.012 | -0.015 | 0.9201 |
| HLADRB | 24 Mo ALT | 70 | 0.099 | -0.005 | 0.7341 |
| DNAM1 | 24 Mo ALT | 66 | 0.029 | -0.015 | 0.9201 |
| MICA | 24 Mo ALT | 69 | -0.348 | 0.108 | **0.0204** |
| Leptin | 24 Mo ALT | 67 | -0.086 | -0.008 | 0.7341 |
| CCL19 | 24 Mo ALT | 70 | -0.230 | 0.039 | 0.1663 |
| NKG2D | 36 Mo ALT | 68 | -0.117 | -0.001 | 0.5107 |
| HLADRB | 36 Mo ALT | 67 | 0.148 | 0.007 | 0.5107 |
| DNAM1 | 36 Mo ALT | 63 | 0.134 | 0.002 | 0.5107 |
| MICA | 36 Mo ALT | 67 | -0.295 | 0.073 | **0.0919** |
| Leptin | 36 Mo ALT | 65 | 0.061 | -0.012 | 0.7566 |
| CCL19 | 36 Mo ALT | 68 | -0.033 | -0.014 | 0.7912 |

**Supplemental Table 4. Univariate linear regression and correlation analysis depicting the predictive power of CCL19 and NKG2D post liver transplantation.** Adj P, adjusted P value based on the false discovery rate according to the Benjamini-Hochberg method considering all analyzed genes; Adj R2, coefficient of determination (adjusted R2) multivariate R; d graft function; AST, aspartate aminotransferase; ALT, alanine transaminase; GGT, gamma-glutamyl transpeptidase; Bili, Bilirubin at discharge, 3, 6 12, 24 and 36 months after transplantation; Stand coeff, standardized coefficient in the respective regression model. P values and adjusted P values < 0.05 are in bold.

|  |  |  | Spearman rank correlation | | |  |
| --- | --- | --- | --- | --- | --- | --- |
| gene | **parameter** | **n** | **stand. coef** | **ρ** | ***P*** | **Adj *P*** |
| CCL19 | 24 Mo BILI | 70 | -0.245 | -0.262 | **0.0282** | 0.1691 |
| CCL19 | 24 Mo AST | 70 | -0.295 | -0.193 | 0.1085 | 0.3255 |
| CCL19 | 24 Mo ALT | 70 | -0.230 | -0.107 | 0.3762 | 0.6289 |
| CCL19 | 36 Mo BILI | 68 | -0.220 | -0.279 | **0.0213** | 0.1276 |
| NKG2D | 3 Mo BILI | 78 | 0.248 | 0.288 | **0.0107** | **0.0641** |
| NKG2D | 6 Mo ALT | 74 | -0.215 | -0.113 | 0.3384 | 0.6149 |

**Supplemental Table 5. Multivariate linear regression analysis of candidate markers.** Functional parameters were log2-transformed. AST, aspartate aminotransferase; ALT, alanine transaminase; GGT, gamma-glutamyl transpeptidase; Bili, Bilirubin at discharge, 3, 6 12, 24 and 36 months after transplantation; P values < 0.05 are in bold.

|  |  |  | Univariate | | Multivariate | |
| --- | --- | --- | --- | --- | --- | --- |
| Functional parameter | Outcome | n | Coefficient | P | Coefficient | P |
| 0 Mo Bili | Donor sex | 82 | -0.008 | 0.959 | -0.023 | 0.888 |
| 0 Mo Bili | Donor age | 82 | 0.004 | 0.432 | 0.004 | 0.482 |
| 0 Mo Bili | Donor BMI | 82 | 0.006 | 0.777 | 0.002 | 0.948 |
| 0 Mo Bili | CIT | 82 | 0.000 | 0.425 | 0.000 | 0.483 |
| 0 Mo Bili | MICA | 82 | -0.009 | 0.839 | -0.019 | 0.680 |
| 0 Mo AST | Donor sex | 82 | 0.113 | 0.523 | 0.102 | 0.576 |
| 0 Mo AST | Donor age | 82 | 0.002 | 0.774 | 0.001 | 0.841 |
| 0 Mo AST | Donor BMI | 82 | 0.001 | 0.958 | 0.001 | 0.970 |
| 0 Mo AST | CIT | 82 | -0.001 | 0.311 | -0.001 | 0.320 |
| 0 Mo AST | MICA | 82 | -0.032 | 0.532 | -0.041 | 0.442 |
| 0 Mo ALT | Donor sex | 82 | -0.143 | 0.616 | -0.145 | 0.611 |
| 0 Mo ALT | Donor age | 82 | -0.006 | 0.478 | -0.007 | 0.404 |
| 0 Mo ALT | Donor BMI | 82 | 0.013 | 0.748 | 0.012 | 0.770 |
| 0 Mo ALT | CIT | 82 | -0.002 | 0.056 | -0.002 | **0.039** |
| 0 Mo ALT | MICA | 82 | -0.074 | 0.366 | -0.089 | 0.291 |
| 0 Mo GGT | Donor sex | 82 | -0.017 | 0.944 | 0.021 | 0.931 |
| 0 Mo GGT | Donor age | 82 | -0.001 | 0.852 | -0.005 | 0.511 |
| 0 Mo GGT | Donor BMI | 82 | 0.064 | 0.057 | 0.066 | 0.063 |
| 0 Mo GGT | CIT | 82 | -0.001 | 0.107 | -0.001 | 0.155 |
| 0 Mo GGT | MICA | 82 | -0.028 | 0.683 | -0.062 | 0.379 |
| 0 Mo AP | Donor sex | 84 | -0.251 | 0.518 | -0.286 | 0.475 |
| 0 Mo AP | Donor age | 84 | 0.010 | 0.399 | 0.010 | 0.439 |
| 0 Mo AP | Donor BMI | 84 | 0.019 | 0.733 | -0.001 | 0.993 |
| 0 Mo AP | CIT | 84 | 0.000 | 0.822 | 0.000 | 0.930 |
| 0 Mo AP | MICA | 84 | 0.071 | 0.527 | 0.055 | 0.641 |
| 3 Mo Bili | Donor sex | 76 | -0.134 | 0.621 | -0.157 | 0.566 |
| 3 Mo Bili | Donor age | 76 | -0.002 | 0.790 | -0.002 | 0.789 |
| 3 Mo Bili | Donor BMI | 76 | 0.010 | 0.783 | 0.005 | 0.907 |
| 3 Mo Bili | CIT | 76 | -0.001 | 0.104 | -0.002 | 0.092 |
| 3 Mo Bili | MICA | 76 | -0.060 | 0.464 | -0.077 | 0.371 |
| 3 Mo AST | Donor sex | 77 | -0.339 | 0.133 | -0.388 | 0.075 |
| 3 Mo AST | Donor age | 77 | -0.002 | 0.782 | 0.001 | 0.907 |
| 3 Mo AST | Donor BMI | 77 | -0.002 | 0.942 | -0.005 | 0.873 |
| 3 Mo AST | CIT | 77 | -0.001 | 0.058 | -0.002 | **0.021** |
| 3 Mo AST | MICA | 77 | -0.150 | **0.028** | -0.172 | **0.013** |
| 3 Mo ALT | Donor sex | 77 | -0.513 | 0.083 | -0.546 | 0.054 |
| 3 Mo ALT | Donor age | 77 | -0.002 | 0.828 | 0.001 | 0.886 |
| 3 Mo ALT | Donor BMI | 77 | 0.029 | 0.471 | 0.038 | 0.356 |
| 3 Mo ALT | CIT | 77 | -0.001 | 0.201 | -0.001 | 0.118 |
| 3 Mo ALT | MICA | 77 | -0.236 | **0.008** | -0.281 | **0.002** |
| 3 Mo GGT | Donor sex | 77 | -0.079 | 0.835 | -0.108 | 0.771 |
| 3 Mo GGT | Donor age | 77 | 0.001 | 0.897 | 0.005 | 0.637 |
| 3 Mo GGT | Donor BMI | 77 | 0.023 | 0.657 | 0.046 | 0.396 |
| 3 Mo GGT | CIT | 77 | 0.000 | 0.782 | 0.000 | 0.703 |
| 3 Mo GGT | MICA | 77 | -0.287 | **0.011** | -0.329 | **0.006** |
| 3 Mo AP | Donor sex | 77 | 0.042 | 0.856 | 0.011 | 0.961 |
| 3 Mo AP | Donor age | 77 | 0.005 | 0.422 | 0.007 | 0.301 |
| 3 Mo AP | Donor BMI | 77 | 0.013 | 0.667 | 0.019 | 0.572 |
| 3 Mo AP | CIT | 77 | 0.000 | 0.568 | 0.000 | 0.561 |
| 3 Mo AP | MICA | 77 | -0.134 | 0.051 | -0.165 | **0.024** |
| 6 Mo Bili | Donor sex | 72 | 0.146 | 0.642 | 0.120 | 0.709 |
| 6 Mo Bili | Donor age | 72 | 0.010 | 0.314 | 0.010 | 0.357 |
| 6 Mo Bili | Donor BMI | 72 | 0.041 | 0.344 | 0.045 | 0.336 |
| 6 Mo Bili | CIT | 72 | 0.000 | 0.920 | 0.000 | 0.796 |
| 6 Mo Bili | MICA | 72 | -0.044 | 0.640 | -0.077 | 0.438 |
| 6 Mo AST | Donor sex | 72 | -0.163 | 0.473 | -0.202 | 0.368 |
| 6 Mo AST | Donor age | 72 | -0.001 | 0.843 | 0.000 | 0.972 |
| 6 Mo AST | Donor BMI | 72 | 0.010 | 0.750 | 0.018 | 0.579 |
| 6 Mo AST | CIT | 72 | -0.001 | 0.424 | -0.001 | 0.278 |
| 6 Mo AST | MICA | 72 | -0.155 | **0.021** | -0.179 | **0.012** |
| 6 Mo ALT | Donor sex | 72 | -0.485 | 0.093 | -0.545 | **0.038** |
| 6 Mo ALT | Donor age | 72 | -0.006 | 0.522 | -0.001 | 0.865 |
| 6 Mo ALT | Donor BMI | 72 | 0.020 | 0.621 | 0.041 | 0.270 |
| 6 Mo ALT | CIT | 72 | -0.001 | 0.445 | -0.001 | 0.159 |
| 6 Mo ALT | MICA | 72 | -0.316 | **1.49E-04** | -0.359 | **2.74E-05** |
| 6 Mo GGT | Donor sex | 72 | -0.410 | 0.291 | -0.468 | 0.228 |
| 6 Mo GGT | Donor age | 72 | 0.001 | 0.924 | 0.005 | 0.702 |
| 6 Mo GGT | Donor BMI | 72 | 0.033 | 0.535 | 0.049 | 0.378 |
| 6 Mo GGT | CIT | 72 | 0.000 | 0.707 | -0.001 | 0.608 |
| 6 Mo GGT | MICA | 72 | -0.239 | **0.039** | -0.287 | **0.019** |
| 6 Mo AP | Donor sex | 71 | -0.118 | 0.542 | -0.155 | 0.428 |
| 6 Mo AP | Donor age | 71 | 0.008 | 0.175 | 0.009 | 0.151 |
| 6 Mo AP | Donor BMI | 71 | 0.024 | 0.369 | 0.023 | 0.411 |
| 6 Mo AP | CIT | 71 | 0.000 | 0.712 | 0.000 | 0.912 |
| 6 Mo AP | MICA | 71 | -0.054 | 0.355 | -0.083 | 0.170 |
| 12 Mo Bili | Donor sex | 75 | -0.337 | 0.221 | -0.268 | 0.332 |
| 12 Mo Bili | Donor age | 75 | 0.002 | 0.840 | -0.001 | 0.902 |
| 12 Mo Bili | Donor BMI | 75 | 0.085 | **0.023** | 0.090 | **0.028** |
| 12 Mo Bili | CIT | 75 | 0.000 | 0.911 | 0.000 | 0.650 |
| 12 Mo Bili | MICA | 75 | 0.006 | 0.943 | -0.031 | 0.721 |
| 12 Mo AST | Donor sex | 75 | -0.384 | **0.047** | -0.402 | **0.039** |
| 12 Mo AST | Donor age | 75 | 0.002 | 0.753 | 0.003 | 0.567 |
| 12 Mo AST | Donor BMI | 75 | 0.014 | 0.611 | 0.011 | 0.690 |
| 12 Mo AST | CIT | 75 | 0.000 | 0.455 | -0.001 | 0.332 |
| 12 Mo AST | MICA | 75 | -0.097 | 0.104 | -0.119 | 0.052 |
| 12 Mo ALT | Donor sex | 75 | -0.886 | **3.63E-04** | -0.934 | **1.36E-04** |
| 12 Mo ALT | Donor age | 75 | -0.001 | 0.941 | 0.004 | 0.600 |
| 12 Mo ALT | Donor BMI | 75 | -0.001 | 0.980 | -0.009 | 0.798 |
| 12 Mo ALT | CIT | 75 | -0.001 | 0.318 | -0.001 | 0.093 |
| 12 Mo ALT | MICA | 75 | -0.173 | **0.027** | -0.198 | 0.008 |
| 12 Mo GGT | Donor sex | 75 | -0.531 | 0.113 | -0.507 | 0.127 |
| 12 Mo GGT | Donor age | 75 | 0.007 | 0.504 | 0.008 | 0.423 |
| 12 Mo GGT | Donor BMI | 75 | 0.072 | 0.118 | 0.081 | 0.095 |
| 12 Mo GGT | CIT | 75 | 0.000 | 0.907 | 0.000 | 0.835 |
| 12 Mo GGT | MICA | 75 | -0.169 | 0.100 | -0.223 | **0.035** |
| 12 Mo AP | Donor sex | 69 | -0.111 | 0.514 | -0.094 | 0.578 |
| 12 Mo AP | Donor age | 69 | 0.008 | 0.126 | 0.008 | 0.150 |
| 12 Mo AP | Donor BMI | 69 | 0.040 | 0.080 | 0.039 | 0.108 |
| 12 Mo AP | CIT | 69 | 0.000 | 0.930 | 0.000 | 0.808 |
| 12 Mo AP | MICA | 69 | -0.010 | 0.837 | -0.046 | 0.394 |
| 24 Mo Bili | Donor sex | 68 | -0.303 | 0.174 | -0.237 | 0.291 |
| 24 Mo Bili | Donor age | 68 | -0.001 | 0.897 | -0.003 | 0.654 |
| 24 Mo Bili | Donor BMI | 68 | 0.062 | **0.044** | 0.062 | 0.064 |
| 24 Mo Bili | CIT | 68 | -0.001 | 0.322 | -0.001 | 0.404 |
| 24 Mo Bili | MICA | 68 | 0.002 | 0.978 | -0.035 | 0.616 |
| 24 Mo AST | Donor sex | 68 | -0.319 | 0.088 | -0.274 | 0.134 |
| 24 Mo AST | Donor age | 68 | -0.006 | 0.312 | -0.004 | 0.465 |
| 24 Mo AST | Donor BMI | 68 | 0.025 | 0.335 | 0.042 | 0.121 |
| 24 Mo AST | CIT | 68 | 0.000 | 0.988 | 0.000 | 0.791 |
| 24 Mo AST | MICA | 68 | -0.132 | **0.017** | -0.149 | **0.010** |
| 24 Mo ALT | Donor sex | 68 | -0.616 | **0.020** | -0.537 | **0.028** |
| 24 Mo ALT | Donor age | 68 | -0.012 | 0.136 | -0.009 | 0.219 |
| 24 Mo ALT | Donor BMI | 68 | 0.036 | 0.334 | 0.063 | 0.078 |
| 24 Mo ALT | CIT | 68 | -0.001 | 0.585 | -0.001 | 0.289 |
| 24 Mo ALT | MICA | 68 | -0.239 | **0.002** | -0.268 | **0.001** |
| 24 Mo GGT | Donor sex | 68 | -0.315 | 0.343 | -0.236 | 0.477 |
| 24 Mo GGT | Donor age | 68 | -0.009 | 0.375 | -0.007 | 0.463 |
| 24 Mo GGT | Donor BMI | 68 | 0.044 | 0.340 | 0.071 | 0.148 |
| 24 Mo GGT | CIT | 68 | 0.000 | 0.991 | 0.000 | 0.823 |
| 24 Mo GGT | MICA | 68 | -0.181 | 0.066 | -0.208 | **0.046** |
| 36 Mo Bili | Donor sex | 66 | -0.187 | 0.318 | -0.105 | 0.570 |
| 36 Mo Bili | Donor age | 66 | -0.004 | 0.456 | -0.006 | 0.292 |
| 36 Mo Bili | Donor BMI | 66 | 0.056 | **0.026** | 0.070 | **0.011** |
| 36 Mo Bili | CIT | 66 | 0.000 | 0.555 | 0.001 | 0.396 |
| 36 Mo Bili | MICA | 66 | -0.013 | 0.818 | -0.030 | 0.601 |
| 36 Mo AST | Donor sex | 66 | -0.418 | 0.029 | -0.400 | **0.038** |
| 36 Mo AST | Donor age | 66 | -0.005 | 0.349 | -0.004 | 0.474 |
| 36 Mo AST | Donor BMI | 66 | 0.011 | 0.675 | 0.014 | 0.597 |
| 36 Mo AST | CIT | 66 | -0.001 | 0.357 | -0.001 | 0.205 |
| 36 Mo AST | MICA | 66 | -0.093 | 0.108 | -0.099 | 0.095 |
| 36 Mo ALT | Donor sex | 66 | -0.445 | 0.114 | -0.426 | 0.123 |
| 36 Mo ALT | Donor age | 66 | -0.012 | 0.128 | -0.009 | 0.260 |
| 36 Mo ALT | Donor BMI | 66 | -0.018 | 0.639 | -0.004 | 0.909 |
| 36 Mo ALT | CIT | 66 | -0.001 | 0.365 | -0.001 | 0.127 |
| 36 Mo ALT | MICA | 66 | -0.197 | **0.019** | -0.187 | **0.030** |
| 36 Mo GGT | Donor sex | 66 | -0.240 | 0.491 | -0.176 | 0.621 |
| 36 Mo GGT | Donor age | 66 | -0.006 | 0.567 | -0.008 | 0.440 |
| 36 Mo GGT | Donor BMI | 66 | 0.052 | 0.272 | 0.061 | 0.238 |
| 36 Mo GGT | CIT | 66 | -0.001 | 0.403 | -0.001 | 0.409 |
| 36 Mo GGT | MICA | 66 | -0.068 | 0.514 | -0.092 | 0.404 |
